# Effects of Oral Doxofylline and Procaterol on Chronic Obstructive Pulmonary Disease: A Randomized Crossover Study

**DOI:** 10.3390/medsci13020049

**Published:** 2025-04-29

**Authors:** Narongkorn Saiphoklang, Sarawut Panichaporn, Thiravit Siriyothipun, Pitchayapa Ruchiwit

**Affiliations:** Division of Pulmonary and Critical Care Medicine, Department of Internal Medicine, Faculty of Medicine, Thammasat University, Pathum Thani 12120, Thailand; oui20081991@gmail.com (S.P.); thiravit.siri@gmail.com (T.S.); rpitchay@tu.ac.th (P.R.)

**Keywords:** bronchodilator, chronic obstructive pulmonary disease, doxofylline, procaterol

## Abstract

**Background**: Oral bronchodilators may serve as an adjunctive therapy in patients with chronic obstructive pulmonary disease (COPD). This study aimed to evaluate the effects of oral doxofylline and oral procaterol on lung function and clinical symptoms in COPD patients. **Methods**: A crossover randomized controlled trial was conducted in patients with clinically stable COPD. Participants first received either doxofylline or procaterol for 4 weeks, followed by a 1-week washout period. Assessments included the modified Medical Research Council (mMRC) dyspnea scale, COPD assessment test (CAT) scores, and 6-minute walking distance (6MWD). Pulmonary function was evaluated using spirometry with bronchodilator (BD) testing and all adverse events were recorded. **Results**: Twenty patients were randomly assigned to begin treatment with either doxofylline or procaterol. Their mean age was 71.7 ± 9.4 years. After four weeks of treatment, the doxofylline group showed significantly greater improvement in pulmonary function parameters (post-BD peak expiratory flow and post-BD forced expiratory flow 25–75) compared to the procaterol group. However, there were no significant differences in mMRC scores, CAT scores, or 6MWD between the two groups. More neurological adverse events were observed in the doxofylline group compared to the procaterol group (35% vs. 5%, *p* = 0.044). **Conclusions**: Doxofylline improved pulmonary function in COPD patients but did not provide superior functional performance compared to procaterol. Neurological adverse events were more frequently associated with doxofylline. Doxofylline may serve as an adjunctive therapy to enhance pulmonary function in COPD patients, but caution is advised due to its potential side effects.

## 1. Introduction

Chronic obstructive pulmonary disease (COPD) is a common respiratory disorder and the third leading cause of death worldwide [1]. It is a heterogeneous lung condition characterized by chronic respiratory symptoms (dyspnea, cough, sputum production, and/or exacerbations) that cause persistent and often progressive airflow obstruction [1,2]. Pharmacotherapy for stable COPD treatment currently focuses on managing symptoms and preventing exacerbation. Bronchodilator medications, particularly inhaled, long-acting bronchodilators (beta2-agonists and muscarinic antagonists), are the mainstay of therapy for symptomatic COPD patients [1]. Oral medications, including methylxanthines (e.g., theophylline, doxofylline) and beta2-agonists (e.g., salbutamol, procaterol), may also be effective in promoting bronchodilation in COPD treatment [1].

Methylxanthines have stimulant effects and promote bronchodilation. Theophylline relaxes the airway smooth muscle by inhibiting phosphodiesterase (PDE) activity (PDE3, PDE4, and PDE5), but relatively high concentrations are needed to achieve maximal relaxation [3]. Theophylline also activates histone deacetylase (HDAC) activity, thereby suppressing the expression of inflammatory genes [4], resulting in anti-inflammatory effects for COPD treatment. Additionally, it blocks the adenosine receptors, potentially causing side effects that affect the central nervous, cardiovascular, gastrointestinal, and urinary systems. These side effects can include seizures, tremors, nausea, frequent urination, and arrhythmia [5]. Theophylline is also associated with several adverse drug reactions, the most severe being its impact on the cardiovascular system [6]. In cases of overdose, it can cause severe arrhythmia or even cardiac arrest. Theophylline is primarily metabolized in the liver by one or more cytochrome P450 enzymes and has a narrow therapeutic window, with effective plasma concentrations ranging from 10 to 20 mcg/mL [7]. Due to these limitations, doxofylline was developed as an alternative medication. Doxofylline has a structure similar to theophylline but also includes a dioxalane group at position 7, which enhances its selectivity for the adenosine A2A receptor [8]. This selectivity reduces the occurrence of side effects and provides a wider therapeutic window than theophylline. Additionally, doxofylline is not metabolized by cytochrome P450 enzymes, which lowers the risk of drug interactions. Consequently, it has become a safer and increasingly preferred option for treating asthma and COPD [9]. Doxofylline also leads to improvement in pulmonary functions compared with baseline [10].

Procaterol is an oral bronchodilator belonging to the beta2-adrenergic receptor agonist class. Procaterol acts directly on bronchial cells by activating adenyl cyclase to stimulate β-receptors, resulting in the relaxation of airway smooth muscle and subsequent bronchodilation [11]. Additionally, procaterol reduces the aggregation of human fetal lung fibroblast-1 (HFL-1) cells, which affects fibroblast activity and may reduce pulmonary fibrosis [12]. Furthermore, procaterol enhances mucociliary clearance, improving the removal of airway secretions [13].

However, the bronchodilatory effects of doxofylline in comparison to procaterol have not yet been explored in patients with COPD. This study aimed to evaluate and compare their effectiveness in improving airway function and controlling symptoms in this population.

## 2. Materials and Methods

### 2.1. Study Design and Participants

A prospective, randomized crossover study was carried out at the pulmonary outpatient clinic of Thammasat University Hospital, Thailand from March 2024 to October 2024. The inclusion criteria included: (1) patients aged 40 years or older, (2) a smoking history of 10 pack-years or more, and (3) a diagnosis of COPD confirmed by a post-bronchodilator (BD) forced expiratory volume in 1 second (FEV_1_) to forced vital capacity (FVC) ratio of less than 0.7. Exclusion criteria included: (1) any pulmonary diseases, such as asthma, bronchiectasis, or pulmonary fibrosis, (2) an inability to perform spirometry or a 6-minute walk test (6MWT), (3) COPD exacerbation within the 12 weeks prior to study recruitment, (4) the use of oral methylxanthines (theophylline or doxofylline), oral roflumilast, oral salbutamol, or oral procaterol within 1 week prior to study recruitment, (5) any chronic disease, such as uncontrolled cardiac arrhythmia, uncontrolled hypertension (blood pressure > 180/110 mmHg), chronic kidney disease (glomerular filtration rate < 50 mL/min), chronic liver disease (liver enzymes > 1.5 times the upper limit of normal), ongoing cancer treatment, or active hyperthyroidism, (6) pregnancy or lactation, and (7) any condition or illness that, at the discretion of the researcher, could hinder the research or interfere with the study results.

Demographic data, respiratory symptoms, and functional capacity were collected using the modified Medical Research Council (mMRC) dyspnea scale [14], COPD assessment test (CAT) [15], 6-minute walking distance (6MWD), and spirometry data. Baseline medications, including inhaled corticosteroids (ICS), long-acting beta2-agonists (LABA), and long-acting muscarinic antagonists (LAMA), were also recorded.

The Global Initiative for Chronic Obstructive Lung Disease (GOLD) criteria for COPD severity were based on the FEV_1_ value: grade 1 indicates mild COPD (≥80% of predicted value), grade 2 indicates moderate COPD (50–79% of predicted value), and grades 3 and 4 indicate severe and very severe COPD (<50% and <30% of predicted values), respectively [1]. GOLD symptoms and risk were classified into three groups: A, B, and E, depending on the dyspnea score and history of exacerbation [1].

Ethical approval was obtained from the Human Research Ethics Committee of Thammasat University (Medicine), Thailand (IRB No. MTU-EC-IM-0-235/66, COA No.067/2024, date of approval: 29 February 2024), in full compliance with international guidelines, including the Declaration of Helsinki, the Belmont Report, CIOMS Guidelines, and the International Conference on Harmonization’s Good Clinical Practice (ICH-GCP). All methods were performed in accordance with these guidelines and regulations. Written informed consent was obtained from all participants. This study was registered on ClinicalTrials.gov with the number NCT06346691.

### 2.2. Randomization and Intervention

**Figure 1:**
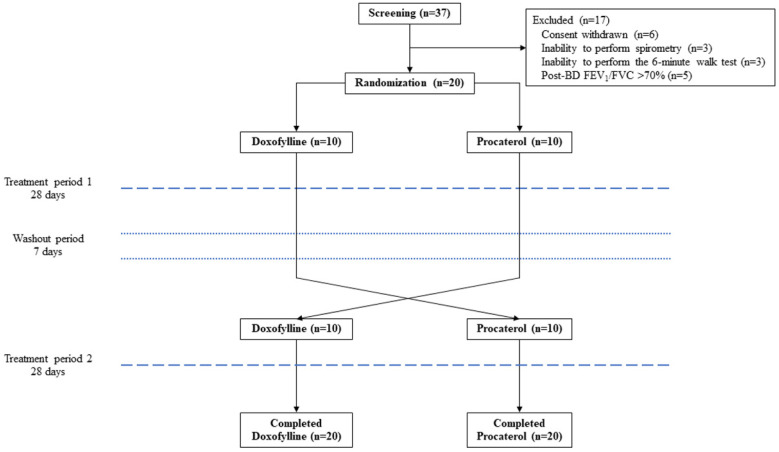
Flow diagram of the prospective, randomized, crossover trial in patients with chronic obstructive pulmonary disease. BD = bronchodilator, FEV_1_ = forced expiratory volume in 1 second, and FVC = forced vital capacity.

Patients were randomly assigned in a 1:1 ratio to receive either doxofylline or procaterol, with randomization performed using a computerized, web-based system. The study flowchart is shown in Figure 1.

Prior to randomization, COPD patients underwent a 1-week run-in period and were then screened for eligibility. Eligible participants were assigned to one of two groups: one received doxofylline at a dose of 400 mg twice daily (800 mg/day), while the other received procaterol at a dose of 50 mcg twice daily (100 mcg/day) for 4 weeks. Following a 1-week washout period, patients crossed over to the alternate treatment for an additional 4 weeks (Figure 1). Study medications were administered by the attending physicians. Throughout the study, all patients underwent spirometry with BD testing, as well as assessments using the mMRC dyspnea scale, CAT, and 6MWT. Adverse events were recorded at the following time points: baseline (visit 0), before the run-in period (visit 1), at the start (visit 2) and end (visit 3) of period 1 treatment, and at the start (visit 4) and end (visit 5) of period 2 treatment. BD testing was conducted using an inhalation of 400 mcg of salbutamol, followed by repeating spirometry after 15 min. Pulmonary function parameters measured by spirometry included FVC, FEV_1_, FEV_1_/FVC, peak expiratory flow (PEF), forced expiration flow rate at 25–75% of FVC (FEF_25–75_), and BD responsiveness. Spirometry was performed in accordance with the American Thoracic Society (ATS) and European Respiratory Society (ERS) guidelines [16,17] using a PC spirometer (Vyntus^®^ SPIRO, Vyaire Medical, Inc., Mettawa, IL, USA).

Doxofylline has a half-life of 7 h [18], and procaterol has a half-life of 3.8 h [19]. Therefore, a washout period of 1 week was sufficient to eliminate the effects of both medications, as this duration exceeds five half-lives for each drug [20].

Before and during the study, patients were allowed to inhale long-acting bronchodilators (beta2-agonists and muscarinic antagonists) for COPD maintenance therapy and inhaled short-acting beta-agonists for symptom relief. On days when spirometry was performed, patients were instructed to take the oral study medications in the morning.

### 2.3. Outcome Assessment

The primary outcome was the difference in pulmonary function parameters between the doxofylline and the procaterol groups following COPD treatment. Secondary outcomes included changes in the pulmonary function parameters, mMRC scores, CAT scores, 6MWD, and the occurrence of adverse events within each group following treatment.

### 2.4. Statistical Analysis

In a study by Wang T and colleagues [21], oral administration of 400 mg of doxofylline per day in COPD patients increased FEV_1_ values by 16.0 ± 7.2% from baseline. In another study by Crowe MJ and colleagues, oral administration of 100 mcg of procaterol in asthmatic patients increased FEV_1_ values by 30.0 ± 10.0% from baseline [22]. We hypothesized that the effects of doxofylline and procaterol on FEV_1_ in COPD patients would align with those observed in previous studies. Based on this hypothesis, we determined that enrolling 20 patients (10 per group) would provide 90% power to detect a difference in pulmonary function, with a two-sided alpha of 0.05.

Categorical data were expressed as a number (%), and continuous data as the mean ± standard deviation (SD). The chi-squared test was used to compare categorical variables between the two groups. The unpaired *t*-test and paired *t*-test were used to compare continuous variables with normal distribution—pulmonary function parameters, mMRC scores, CAT scores, and 6MWD—between the two independent and dependent groups, respectively. The Mann–Whitney U test and Wilcoxon signed-rank test were used to compare continuous variables with non-normal distributions between two independent and dependent groups, respectively. A two-sided *p*-value of <0.05 was considered statistically significant. Statistical analyses were performed using SPSS version 24 software (IBM Corp., Armonk, NY, USA).

## 3. Results

### 3.1. Participants

A total of 37 COPD patients were screened, and 20 eligible patients were subsequently randomized into the study. No participants were lost to follow-up throughout the study duration (Figure 1). The twenty participants were predominantly male (95.0%), with a mean age of 71.7 ± 9.4 years (Table 1). Common comorbidities included hyperlipidemia (80%), hypertension (65%), and cardiovascular disease (40%). Most participants were classified as GOLD grade 2 (60%) and group A (70%). Triple therapy (ICS/LABA/LAMA) was the predominant treatment (45%). The mean mMRC score was 1.1 ± 0.9, the CAT score was 6.3 ± 5.6, and the 6MWD was 391.9 ± 107.7 m. Post-BD FEV_1_ performance was 59.34 ± 13.92% of the predicted value (Table 1).

### 3.2. Primary Outcome

Compared to the procaterol group, the doxofylline group showed significantly greater improvement in pulmonary function parameters: post-BD PEF value (mean difference 0.455, 95% CI 0.127 to 0.782 L/s, *p* = 0.008), post-BD FEF_25–75_ value (mean difference 0.083, 95% CI 0.023 to 0.143 L/s, *p* = 0.008), and post-BD FEF_25–75_% predicted (mean difference 4.115, 95% CI 0.917 to 7.313% predicted, *p* = 0.013). However, there were no significant differences in other pulmonary function parameters, mMRC scores, CAT scores, or 6MWD between the two groups (Table 2).

### 3.3. Secondary Outcomes

After the 4-week treatment, there were significant increases in post-BD FEF_25–75_ value of 0.041 ± 0.082 L/s (*p* = 0.040) and post-BD FEF_25–75_ percentage of 2.310 ± 4.268% predicted (*p* = 0.026) in the doxofylline group. Conversely, significant decreases were observed in the procaterol group, with a post-BD PEF value of 0.352 ± 0.568 L/s (*p* = 0.015) and a percentage of PEF change after BD test of 5.884 ± 11.900% (*p* = 0.045) (Table 3). However, there were no significant changes in other pulmonary function parameters, mMRC scores, CAT scores, or 6MWD in either group after the 4-week treatment (Table 3).

Neurological adverse events were observed more markedly in the doxofylline group compared to the procaterol group (35% vs. 5%, *p* = 0.044), particularly regarding dizziness (25% vs. 0%, *p* = 0.047) (Table 4). A serious adverse event, i.e., one death, was reported in the procaterol group.

## 4. Discussion

This study is the first crossover randomized trial comparing the bronchodilatory effects of these two oral bronchodilators in COPD. We found that patients in the doxofylline group showed significantly greater improvement in pulmonary function parameters (post-BD PEF and post-BD FEF_25–75_) compared to those in the procaterol group after the 4-week treatment. However, neurological side effects were significant in the doxofylline group, and one patient in the procaterol group died during the study.

According to GOLD recommendations [1], inhaled bronchodilators are the mainstay treatment of COPD to prevent exacerbation and control the respiratory symptoms. Oral bronchodilators may be an alternative option for COPD treatment, particularly in cases of inhalation technique error, poor hand–breath coordination, insufficient inspiratory effort, or uncontrolled COPD despite the use of maximum doses of inhaled medications. A meta-analysis by Cho-Reyes S et al. found that 86.7% of adults with obstructive lung diseases using metered dose inhalers exhibited at least one inhalation technique error, and 76.9% incorrectly performed ≥ 20% of the device use steps [23]. Critical errors in inhalation were associated with worse clinical outcomes in patients with asthma or COPD, as reviewed by Kocks JWH et al. [24]. In addition, suboptimal peak inspiratory flow and inhalation technique errors were related to higher COPD-related healthcare utilization and costs in COPD patients using dry powder inhalers for maintenance therapy, as found in a study by Leving MT et al. [25]. Oral bronchodilators improve COPD symptoms and quality of life in COPD patients.

Methylxanthines exhibit stimulatory effects, are able to cause bronchodilation, and possess anti-inflammatory properties [3,4,26]. Theophylline, a methylxanthine, has been shown to significantly reduce histone deacetylase-2 levels in macrophages and the peripheral lung tissue of COPD patients, leading to decreased inflammation and reduced steroid resistance [26]. In a randomized controlled trial by Murciano D et al. [27], theophylline improved respiratory function and alleviated dyspnea in patients with severe COPD. Additionally, a randomized, double-blind, placebo-controlled study by Zhou Y et al. [28] found that theophylline led to greater improvements in pre-BD FEV_1_, a lower frequency of COPD exacerbations, fewer days of exacerbation, less frequent clinical visits, and enhanced quality of life compared to the placebo group. Furthermore, a study by Bellia V et al. [29] demonstrated that theophylline improved FEV_1_, PEF, and symptoms in COPD patients, showing similar efficacy to inhaled oxitropium bromide or a combination of both drugs.

Doxofylline, which differs from theophylline by containing a dioxalane group at position 7, demonstrates comparable efficacy to theophylline in treating respiratory diseases. However, unlike theophylline, it offers an improved tolerability profile, a more favorable risk-to-benefit ratio, and no significant drug–drug interactions [8]. Therefore, doxofylline is considered a safer and more favorable option for the treatment of COPD and asthma [9]. Moreover, doxofylline exhibits significant anti-inflammatory activity in the lungs, which can lead to a notable steroid-sparing effect [30]. A study by Lal D et al. [31] showed that both theophylline and doxofylline improved pulmonary function (FEV_1_, FVC, and FEV_1_/FVC) at different time intervals in asthma and COPD patients. The maximum effects were observed at 6 weeks for asthma patients and at 8 weeks for COPD patients for both drugs. Doxofylline showed a trend toward fewer side effects compared to theophylliWang T et al. [21] reported that both oral doxofylline (200 mg twice daily) and inhaled tiotropium (18 μg/day) significantly improved pulmonary function (FEV_1_ and FEV_1_/FVC%) from baseline at 12 and 24 weeks in patients with moderate to severe but stable COPD. However, major adverse effects occurred in 12.9% of the doxofylline group, compared to 9.9% of the tiotropium group. Furthermore, a meta-analysis by Cazzola M et al. [10], which included 20 studies involving 820 COPD patients, found that doxofylline improved FEV_1_ by 8.2% and 317 mL over baseline. However, patients treated with doxofylline treatment experienced more side effects, such as epigastric discomfort, nausea, and headaches, compared to the placebo group.

Our study demonstrated that doxofylline significantly improved only the FEF_25–75_ value, indicating an effect on small airway disease, but not on FEV_1_ or FVC. This may be because most of our patients had baseline pulmonary function that was classified as mild to moderate rather than severe, making it difficult to observe significant changes from baseline. Similarly, the baseline COPD symptoms, assessed by the mMRC and CAT scores in our study, indicated mild symptoms (mean mMRC score of 1.1 and CAT score of 6.3), which also made it challenging to detect changes in symptoms from baseline. Moreover, the 4-week duration of doxofylline treatment in our study may have been too short to achieve the maximum therapeutic effects of treatment, which, in a study by Lal D et al. [31], required at least 8 weeks. Consequently, FEV_1_ and FVC, which reflect larger airway function, did not show significant changes after the 4-week treatment period.

A positive bronchodilator response is typically defined as an increase of at least 12% and an absolute value of at least 200 mL compared to the baseline in either FEV_1_ or FVC [32]. However, our results did not meet this criterion. Additionally, to accurately compare differences in bronchodilation—whether between the start and end of treatment or among different medications—it is essential to evaluate not only the statistical differences but also the minimal clinically important difference (MCID) in the clinical parameters, including lung function (an increase in FEV_1_ of 100 mL), exercise capacity (a 6MWD increase of 26 m), and respiratory symptoms (a two-point change in CAT) [33,34,35]. In our study, changes in these clinical outcomes did not reach their MCIDs.

Procaterol, a beta2-adrenergic receptor agonist, is a bronchodilator used in the treatment of COPD and asthma. It works by directly relaxing the airway smooth muscles, resulting in bronchodilation [36]. A study by Sukisaki T et al. [37] demonstrated that, in patients with moderate to severe COPD, the inhalation of 20 μg of procaterol significantly increased walking distance at 4 h post-inhalation compared to the no-treatment group (331 ± 119 m vs. 294 ± 113 m, *p* < 0.001), despite there being no significant difference in FEV_1_ between the groups. However, these findings were not observed in our study involving 4 weeks of oral procaterol treatment. A study by Shioya T et al. [38] revealed that, when compared to inhaled oxitropium (200 μg thrice daily), inhaled procaterol (20 μg thrice daily) significantly improved pulmonary function (FEV_1_, FVC, total lung capacity, functional residual capacity, residual volume, maximal inspiratory pressure, and maximal expiratory pressure), 6MWD, and the Borg dyspnea scale, as well as scores for dyspnea, fatigue, emotional function, mastery, total scores, and activities of daily living over 12, 24, and 52 weeks compared to baseline. However, no clinical studies have been published on the use of oral procaterol in COPD patients. Our study is the first clinical study of the effect of oral procaterol in COPD patients.

Although our COPD patients showed improvement in pulmonary function after 4 weeks of oral bronchodilator treatment, some adverse events were observed during the study. Neurological adverse events were more commonly observed in the doxofylline group (35%), particularly regarding dizziness (25%). These side effects may lead to the discontinuation of doxofylline in 20% of patients. A serious adverse event (one death) was reported in the procaterol group. The case involved an 88-year-old female with hypertension, atrial fibrillation, and COPD. She completed the first 4-week period of study with doxofylline but experienced a cardiac arrest at night after taking oral procaterol for 2 days. The cause of death could not be determined, as her family member did not request an autopsy to identify the cause. Therefore, we suggest that elderly COPD patients with underlying cardiovascular disease, particularly arrhythmia, should either avoid using or be closely monitored for serious side effects when using oral bronchodilators for COPD treatment.

Additionally, our study found newly emerging COPD exacerbation during the study period in both groups, and treatment was discontinued in the doxofylline group. This may have resulted from the lower effectiveness of medications in reducing COPD exacerbation, along with a higher rate of side effects.

This study has several limitations. Firstly, the sample size was small, with a mean age of 71 years and only 5% of participants being female. This limited sample may not be representative of the broader clinical population. Secondly, our patients had well-controlled COPD symptoms at the time of study enrollment. As a result, the findings may not show significant differences in other pulmonary function parameters, CAT scores, mMRC scores, or 6MWD after oral bronchodilator treatments. Thirdly, a single- or double-blind design could not be implemented due to differences in the packaging of both medications. Consequently, both physicians and patients could identify the study drugs, potentially leading to bias in the clinical outcomes. To mitigate this, we employed a cross-over design. Lastly, the 4-week follow-up period may have been too brief to capture meaningful changes in pulmonary function or clinical outcomes, especially COPD exacerbation. To more thoroughly assess the effectiveness of oral bronchodilators, larger-scale studies with longer follow-up durations are needed.

## 5. Conclusions

Doxofylline treatment demonstrated improvements in pulmonary function in COPD patients; however, it did not result in superior functional performance compared to procaterol. Notably, neurological adverse events, such as dizziness, were more frequently associated with doxofylline treatment, highlighting the potential risks of this medication. Despite these concerns, doxofylline may still serve as an adjunctive therapy to enhance pulmonary function in COPD patients. However, its use should be approached with caution, particularly in patients at higher risk of adverse events, and careful monitoring is recommended to ensure patient safety.

## Figures and Tables

**Table 1:** Baseline characteristics of COPD patients.

Characteristics	Total (n = 20)	Doxofylline (n = 10)	Procaterol (n = 10)	*p*-Value
Age, years	71.7 ± 9.4	74.1 ± 9.4	69.3 ± 9.1	0.263
Male/female	19 (95)/1 (5)	9 (90)/1 (10)	10 (100)/0 (0)	1.000
Body mass index, kg/m^2^	23.2 ± 4.2	22.5 ± 4.0	23.8 ± 4.5	0.487
Formerly smoking	16 (80)	9 (90)	7 (70)	0.453
Smoking, pack-years	38.0 ± 22.8	34.6 ± 23.0	41.4 ± 23.2	0.519
Comorbidity				
Dyslipidemia	16 (80)	9 (90)	7 (70)	0.582
Hypertension	13 (65)	8 (80)	5 (50)	0.350
Coronary artery disease	8 (40)	5 (50)	3 (30)	0.980
Diabetes mellitus	7 (35)	4 (40)	3 (30)	1.000
Obstructive sleep apnea	4 (20)	2 (20)	2 (20)	1.000
Chronic kidney disease	4 (20)	3 (30)	1 (10)	0.582
Allergic rhinitis	7 (35)	4 (40)	3 (30)	1.000
COPD grade				0.478
1	1 (5)	1 (10.0)	0 (0)	
2	12 (60)	5 (50)	7 (70)	
3	7 (35)	4 (40)	3 (30)	
COPD group				0.549
A	14 (70)	7 (70)	7 (70)	
B	5 (25)	3 (30)	2 (20)	
E	1 (5)	0 (0)	1 (10)	
Medication				0.736
LAMA	1 (5)	1 (10)	0 (0)	
LABA/LAMA	8 (40)	4 (40)	4 (40)	
ICS/LABA	2 (10)	1 (10)	1 (10)	
ICS/LABA/LAMA	9 (45)	4 (40)	5 (40)	
Functional capacity				
mMRC scores	1.1 ± 0.9	1.3 ± 0.9	0.6 ± 1.0	0.119
CAT scores	6.3 ± 5.6	6.2 ± 4.4	5.0 ± 8.3	0.689
6MWD, m	391.9 ± 107.7	357.0 ± 102.7	432.9 ± 101.5	0.114
Laboratory data				
Blood eosinophils, %	3.57 ± 2.70	4.40 ± 3.51	2.73 ± 1.25	0.183
Blood eosinophil counts, cells/mm^3^	250.20 ± 178.09	276.9 ± 224.8	223.5 ± 121.9	0.518
Blood eosinophil counts ≥ 300 cells/mm^3^	14 (35)	7 (70)	7 (70)	1.000
Spirometry data				
Pre-BD FVC, L	2.68 ± 0.69	2.42 ± 0.80	2.98 ± 0.51	0.078
Pre-BD FVC, % predicted	86.11 ± 16.77	82.62 ± 20.62	91.63 ± 16.67	0.297
Post-BD FVC, L	2.84 ± 0.73	2.50 ± 0.82	3.07 ± 0.53	0.084
Post-BD FVC, % predicted	91.33 ± 17.45	85.61 ± 20.70	94.35 ± 18.09	0.328
FVC change after BD test, %	3.37 ± 4.54	3.92 ± 4.53	2.82 ± 4.72	0.601
Pre-BD FEV_1_, L	1.35 ± 0.39	1.36 ± 0.46	1.34 ± 0.34	0.886
Pre-BD FEV_1_, % predicted	55.56 ± 14.06	59.74 ± 14.80	52.40 ± 14.20	0.273
Post-BD FEV_1_, L	1.41 ± 0.36	1.42 ± 0.46	1.38 ± 0.33	0.837
Post-BD FEV_1_, % predicted	59.34 ± 13.92	61.74 ± 14.18	51.11 ± 18.37	0.165
FEV_1_ change after BD test, %	3.72 ± 5.75	3.77 ± 6.35	3.66 ± 5.42	0.967
Pre-BD FEV_1_/FVC, %	50.39 ± 11.39	57.78 ± 11.21	45.31 ± 10.57	0.020
Post-BD FEV_1_/FVC, %	51.17 ± 12.49	57.55 ± 10.61	45.76 ± 11.08	0.026
FEV_1_/FVC change after BD test, %	0.39 ± 4.41	−0.08 ± 4.85	0.86 ± 4.11	0.646
Pre-BD PEF, L/s	4.37 ± 1.26	4.93 ± 1.63	4.21 ± 0.99	0.249
Pre-BD PEF, % predicted	59.96 ± 15.86	70.88 ± 19.77	56.87 ± 13.79	0.083
Post-BD PEF, L/s	4.61 ± 1.25	4.99 ± 1.67	4.32 ± 0.87	0.276
Post-BD PEF, % predicted	63.46 ± 15.37	71.15 ± 19.83	58.43 ± 12.81	0.106
PEF change after BD test, %	2.15 ± 12.76	0.24 ± 10.88	4.05 ± 14.75	0.519
Pre-BD FEF_25–75_, L/s	0.49 ± 0.25	0.58 ± 0.29	0.39 ± 0.17	0.115
Pre-BD FEF_25–75_, % predicted	20.60 ± 10.25	29.90 ± 14.76	18.43 ± 8.46	0.047
Post-BD FEF_25–75_, L/s	0.51 ± 0.24	0.52 ± 0.23	0.44 ± 0.18	0.397
Post-BD FEF_25–75_, % predicted	22.83 ± 10.37	25.94 ± 9.71	20.50 ± 9.59	0.223
FEF_25–75_ change after BD test, %	2.08 ± 20.31	−7.08 ± 22.54	11.23 ± 13.27	0.040

Data shown as n (%) or mean ± SD, 6MWD = 6-minute walking distance, BD = bronchodilator, CAT = COPD assessment test, COPD = chronic obstructive pulmonary disease, FEF_25–75_ = forced expiratory flow at 25–75% of FVC, PEF = peak expiratory flow, FEV_1_ = forced expiratory volume in 1 second, FVC = forced vital capacity, ICS = inhaled corticosteroids, kg = kilogram, L = liter, LABA = long-acting beta2-agonists, LAMA = long-acting muscarinic antagonists, m = meter, mm = millimeter, mMRC = modified Medical Research Council value, s = second.

**Table 2:** Changes in spirometry data and functional capacity of COPD patients after 4 weeks of doxofylline and procaterol treatment.

Data	Doxofylline	Procaterol	Mean Difference (95% CI)	*p*-Value
Spirometry data change from baseline				
Pre-BD FVC, L	−0.021 ± 0.224	−0.021 ± 0.196	0.000 (−0.137, 0.137)	1.000
Pre-BD FVC, % predicted	−0.365 ± 8.995	−1.011 ± 6.858	0.645 (−4.565, 5.856)	0.803
Post-BD FVC, L	−0.195 ± 0.254	−0.073 ± 0.222	0.054 (−0.101, 0.209)	0.488
Post-BD FVC, % predicted	−0.580 ± 10.126	−2.874 ± 7.490	2.294 (−3.510, 8.098)	0.428
FVC change after BD test, %	−0.435 ± 6.551	−2.258 ± 9.440	1.823 (−3.426, 7.072)	0.486
Pre-BD FEV_1_, L	−0.001 ± 0.128	−0.015 ± 0.112	0.724 (−0.065, 0.092)	0.724
Pre-BD FEV_1_, % predicted	0.160 ± 6.623	−0.847 ± 4.758	1.007 (−2.752, 4.766)	0.590
Post-BD FEV_1_, L	0.004 ± 0.116	−0.031 ± 0.150	0.035 (−0.052, 0.121)	0.426
Post-BD FEV_1_, % predicted	1.750 ± 7.643	−1.642 ± 5.762	3.392 (−1.018, 7.802)	0.128
FEV_1_ change after BD test, %	0.190 ± 5.624	−1.421 ± 8.088	1.611 (−2.889, 6.111)	0.473
Pre-BD FEV_1_/FVC, %	0.207 ± 3.074	−0.343 ± 2.927	0.550 (−1.399, 2.500)	0.571
Post-BD FEV_1_/FVC, %	0.565 ± 2.594	0.381 ± 3.754	0.183 (−1.901, 2.268)	0.859
FEV_1_/FVC change after BD test, %	0.710 ± 4.670	1.089 ± 9.490	−0.379 (−5.194, 4.435)	0.874
Pre-BD PEF, L/s	0.127 ± 0.495	−0.070 ± 0.563	0.197 (−0.147, 0.541)	0.253
Pre-BD PEF, % predicted	2.125 ± 7.948	−4.979 ± 20.830	7.104 (−3.026, 17.233)	0.164
Post-BD PEF, L/s	0.103 ± 0.436	−0.352 ± 0.568	0.455 (0.127, 0.782)	0.008
Post-BD PEF, % predicted	−0.215 ± 12.637	−4.663 ± 7.949	4.448 (−2.444, 11.340)	0.199
PEF change after BD test, %	−1.330 ± 15.503	−5.884 ± 11.900	4.554 (−4.448, 13.556)	0.312
Pre-BD FEF_25–75_, L/s	0.011 ± 0.144	0.001 ± 0.072	0.010 (−0.064, 0.085)	0.778
Pre-BD FEF_25–75_, % predicted	0.670 ± 8.266	0.274 ± 3.573	0.396 (−3.775, 4.568)	0.848
Post-BD FEF_25–75_, L/s	0.041 ± 0.082	−0.426 ± 0.102	0.083 (0.023, 0.143)	0.008
Post-BD FEF_25–75_, % predicted	2.310 ± 4.268	−1.805 ± 5.538	4.115 (0.917, 7.313)	0.013
FEF_25–75_ change after BD test, %	2.840 ± 22.101	−9.021 ± 31.336	11.861 (−5.659, 29.381)	0.178
Functional capacity				
mMRC scores	−0.100 ± 0.447	−0.105 ± 0.315	0.005 (−0.247, 0.258)	0.967
CAT scores	−1.000 ± 4.316	−0.473 ± 2.458	−0.526 (−2.822, 1.769)	0.645
6MWD, m	7.450 ± 30.705	9.053 ± 28.905	−1.603 (−20.974, 17.769)	0.868

Data shown as mean ± SD, 6MWD = 6-minute walking distance, BD = bronchodilator, CAT = COPD assessment test, COPD = chronic obstructive pulmonary disease, FEF_25–75_ = forced expiratory flow at 25–75% of FVC, PEF = peak expiratory flow, FEV_1_ = forced expiratory volume in 1 second, FVC = forced vital capacity, L = liters, m = meter, mMRC = modified Medical Research Council, s = second.

**Table 3:** Pulmonary function and functional performance of COPD patients after 4 weeks of doxofylline and procaterol treatment.

Data	Doxofylline				Procaterol			
	Before Treatment	After Treatment	Mean Change (95% CI)	*p*-Value	Before Treatment	After Treatment	Mean Change (95% CI)	*p*-Value
Pre-BD FVC, L	2.699 ± 0.717	2.679 ± 0.645	−0.021 ± 0.224 (−0.125, 0.084)	0.687	2.678 ± 0.643	2.658 ± 0.636	−0.021 ± 0.196 (−0.115, 0.074)	0.654
Pre-BD FVC, % predicted	87.125 ± 18.828	86.760 ± 17.225	−0.365 ± 8.995 (−4.575, 3.845)	0.858	84.858 ± 20.159	83.847 ± 19.200	−1.011 ± 6.858 (−4.316, 2.295)	0.529
Post-BD FVC, L	2.784 ± 0.733	2.764 ± 0.714	−0.195 ± 0.254 (−0.138, 0.099)	0.735	2.806 ± 0.649	2.733 ± 0.659	−0.073 ± 0.222 (−0.180, 0.034)	0.168
Post-BD FVC, % predicted	89.980 ± 19.447	89.400 ± 18.636	−0.580 ± 10.126 (−5.319, 4.159)	0.801	88.779 ± 19.603	85.905 ± 18.647	−2.874 ± 7.490 (−6.484, 0.737)	0.112
FVC change after BD test, %	3.370 ± 4.536	2.935 ± 5.318	−0.435 ± 6.551 (−3.501, 2.631)	0.770	5.268 ± 8.125	3.011 ± 8.398	−2.258 ± 9.440 (−6.808, 2.292)	0.311
Pre-BD FEV_1_, L	1.351 ± 0.390	1.350 ± 0.362	−0.001 ± 0.128 (−0.061, 0.059)	0.973	1.336 ± 0.381	1.322 ± 0.393	−0.015 ± 0.112 (−0.069, 0.039)	0.572
Pre-BD FEV_1_, % predicted	56.070 ± 14.611	56.230 ± 14.643	0.160 ± 6.623 (−2.939, 3.259)	0.915	54.068 ± 15.817	53.221 ± 15.756	−0.847 ± 4.758 (−3.141, 1.446)	0.448
Post-BD FEV_1_, L	1.397 ± 0.386	1.401 ± 0.374	0.004 ± 0.116 (−0.050, 0.058)	0.879	1.435 ± 0.356	1.404 ± 0.366	−0.031 ± 0.150 (−0.103, 0.042)	0.387
Post-BD FEV_1_, % predicted	56.425 ± 16.875	58.175 ± 14.151	1.750 ± 7.643 (−1.827, 5.327)	0.319	58.116 ± 14.782	56.474 ± 14.211	−1.642 ± 5.762 (−4.419,1.135)	0.230
FEV_1_ change after BD test, %	3.715 ± 5.748	3.905 ± 4.069	0.190 ± 5.624 (−2.442, 2.822)	0.881	8.900 ± 9.453	7.479 ± 9.161	−1.421 ± 8.088 (−5.320, 2.477)	0.454
Pre-BD FEV_1_/FVC, %	51.546 ± 12.386	51.753 ± 12.487	0.207 ± 3.074 (−1.231, 1.645)	0.767	51.181 ± 13.692	50.837 ± 12.927	−0.343 ± 2.927 (−1.754, 1.068)	0.616
Post-BD FEV_1_/FVC, %	51.650 ± 12.167	52.215 ± 12.427	0.565 ± 2.594 (−0.650, 1.779)	0.343	52.658 ± 13.026	53.040 ± 13.700	0.381 ± 3.754 (−1.428, 2.191)	0.663
FEV_1_/FVC change after BD test, %	0.390 ± 4.406	1.100 ± 4.752	0.710 ± 4.670 (−1.476, 2.896)	0.505	3.542 ± 5.847	4.632 ± 8.147	1.089 ± 9.490 (−3.485, 5.663)	0.623
Pre-BD PEF, L/s	4.573 ± 1.367	4.700 ± 1.113	0.127 ± 0.495 (−0.105, 0.359)	0.266	4.620 ± 1.182	4.550 ± 1.180	−0.070 ± 0.563 (−0.342, 0.202)	0.595
Pre-BD PEF, % predicted	63.875 ± 18.079	66.000 ± 14.486	2.125 ± 7.948 (−1.595, 5.845)	0.247	63.274 ± 15.999	58.295 ± 18.752	−4.979 ± 20.830 (−15.019, 5.061)	0.311
Post-BD PEF, L/s	4.652 ± 1.343	4.7545 ± 1.258	0.103 ± 0.436 (−0.102, 0.307)	0.307	4.897 ± 1.080	4.545 ± 0.958	−0.352 ± 0.568 (−0.626, −0.078)	0.015
Post-BD PEF, % predicted	64.790 ± 17.511	64.575 ± 15.294	−0.215 ± 12.637 (−6.129, 5.699)	0.940	67.137 ± 14.740	62.474 ± 12.719	−4.663 ± 7.949 (−8.495, −0.832)	0.020
PEF change after BD test, %	2.145 ± 12.762	0.815 ± 9.176	−1.330 ± 15.503 (−8.586, 5.926)	0.705	7.321 ± 11.070	1.437 ± 9.632	−5.884 ± 11.900 (−11.620, −0.149)	0.045
Pre-BD FEF_25–75,_ L/s	0.486 ± 0.250	0.497 ± 0.223	0.011 ± 0.144 (−0.057, 0.079)	0.074	0.466 ± 0.237	0.466 ± 0.252	0.001 ± 0.072 (−0.034, 0.035)	0.975
Pre-BD FEF_25–75,_ % predicted	24.160 ± 13.105	24.830 ± 11.553	0.670 ± 8.266 (−3.199, 4.539)	0.721	22.268 ± 12.059	22.542 ± 13.056	0.274 ± 3.573 (−1.448, 1.996)	0.742
Post-BD FEF_25–75,_ L/s	0.476 ± 0.205	0.517 ± 0.226	0.041 ± 0.082 (0.002, 0.079)	0.040	0.532 ± 0.233	0.490 ± 0.238	−0.426 ± 0.102 (−0.092, 0.007)	0.086
Post-BD FEF_25–75,_ % predicted	23.220 ± 9.795	25.530 ± 11.218	2.310 ± 4.268 (0.313, 4.307)	0.026	25.374 ± 11.638	23.568 ± 11.563	−1.805 ± 5.538 (−4.474, 0.864)	0.172
FEF_25–75_ change after BD test, %	2.075 ± 20.309	4.915 ± 14.577	2.840 ± 22.101 (−7.504, 13.184)	0.572	18.700 ± 18.752	9.679 ± 23.757	−9.021 ± 31.336 (−24.124, 6.082)	0.226
mMRC scores	0.950 ± 0.999	0.850 ± 0.875	−0.100 ± 0.447 (−0.309, 0.109)	0.330	1.000 ± 0.882	0.890 ± 0.937	−0.105 ± 0.315 (−0.257, 0.047)	0.163
CAT scores	5.600 ± 6.460	4.600 ± 5.605	−1.000 ± 4.316 (−3.020, 1.020)	0.313	5.320 ± 5.239	4.840 ± 6.423	−0.474 ± 2.458 (−1.658, 0.711)	0.412
6MWD, m	394.950 ± 106.729	402.400 ± 96.960	7.450 ± 30.705 (−6.920, 21.820)	0.291	408.737 ± 108.588	417.790 ± 112.749	9.053 ± 28.905 (−4.879, 22.984)	0.189

Data shown as mean ± SD, 6MWD = 6-minute walking distance, BD = bronchodilator, CAT = COPD assessment test, COPD = chronic obstructive pulmonary disease, FEF_25–75_ = forced expiratory flow at 25–75% of FVC, PEF = peak expiratory flow, FEV_1_ = forced expiratory volume in 1 second, FVC = forced vital capacity, L = liter, m = meter, mMRC = modified Medical Research Council, s = second.

**Table 4:** Adverse events of doxofylline and procaterol treatment in COPD patients.

Adverse Events	Doxofylline (n = 20)	Procaterol (n = 20)	*p*-Value
Serious adverse event	0	1 (5)	1.000
COPD exacerbation	1 (5)	2 (10)	1.000
Stopping medication	4 (20)	0	0.106
Cardiovascular adverse events	3 (15)	1 (5)	0.605
Palpitation	3 (15)	1 (5)	0.605
Tachycardia	0	0	NA
Chest pain	1 (5)	0	1.000
Gastrointestinal adverse events	5 (25)	1 (5)	0.182
Nausea	5 (25)	1 (5)	0.182
Diarrhea	1 (5)	0	1.000
Abdominal pain	1 (5)	0	1.000
Anorexia	3 (15)	0	0.231
Neurological adverse events	7 (35)	1 (5)	0.044
Headache	0	0	NA
Dizziness	5 (25)	0	0.047
Insomnia	3 (15)	1 (5)	0.605
Respiratory adverse events	2 (10)	3 (15)	1.000
Rhinitis	0	1 (5)	1.000
Pharyngitis	0	0	NA
Increased cough	0	0	NA
Dry mouth	2 (10)	0	0.487

Data shown as n (%), mean ± SD, COPD = chronic obstructive pulmonary disease, NA = not applicable.

## Data Availability

The data supporting the results of this study are available within the article.

## Abbreviations

The following abbreviations are used in this manuscript:

ATS	American Thoracic Society
BD	bronchodilator
CAT	COPD assessment test
COPD	chronic obstructive pulmonary disease
ERS	European Respiratory Society
FEF_25–75_	forced expiration flow rate at 25–75% of FVC
FEV_1_	forced expiratory volume in 1 second
FVC	forced vital capacity
GOLD	Global Initiative for Chronic Obstructive Lung Disease
HDAC	histone deacetylase
HFL-1	human fetal lung fibroblasts-1
ICS	inhaled corticosteroids
LABA	long-acting beta-2 agonists
LAMA	long-acting muscarinic antagonists
MCID	minimal clinically important difference
mMRC	modified Medical Research Council
PDE	phosphodiesterase
PEF	peak expiratory flow
6MWD	6-minute walking distance
6MWT	6-minute walk test
